# Effects of Immunologically Active Peptides on Growth Performance, Meat Quality, Antioxidant Capacity, and Ileum Inflammation in Broilers

**DOI:** 10.3390/vetsci13090870

**Published:** 2026-08-26

**Authors:** Zhiwen Bian, Ruyi Han, Luca Marchetti, Shixin Li, Yi Zou, Guido Invernizzi, Haijun Zhang, Valentino Bontempo, Chao Jian, Xianren Jiang

**Affiliations:** 1 Key Laboratory of Feed Biotechnology of Ministry of Agriculture and Rural Affairs, Institute of Feed Research, Chinese Academy of Agricultural Sciences, Beijing 100081, China; bianzhiwen2024@163.com (Z.B.); hhhry5201314@163.com (R.H.); 18689594083@163.com (Y.Z.); zhanghaijun@caas.cn (H.Z.); 2 Department of Veterinary Medicine and Animal Science (DIVAS), University of Milan, 26900 Lodi, Italy; luca.marchetti1@unimi.it (L.M.); guido.invernizzi@unimi.it (G.I.); valentino.bontempo@unimi.it (V.B.); 3 Shandong Kunhe Xinchuang Bioengineering Co., Ltd., Zaozhuang 277000, China; lsx.138@163.com (S.L.); jianchao886699@163.com (C.J.)

**Keywords:** poultry, feed additive, growth performance, nutrition, antioxidant capacity, gut health

## Abstract

Immunologically active peptides (IAP) are important bioactive peptides with potential antioxidant, anti-inflammatory, and immunomodulatory properties. However, their application in broiler production has not been systematically evaluated. This study investigated the effects of dietary IAP supplementation on growth performance, carcass traits, meat quality, selected serum biochemical indices, and ileum inflammatory cytokine expression in broilers. A total of 180 one-day-old Ross 308 male broilers were randomly assigned to 3 dietary treatments with 6 replicates per group for 42 d: a basal diet (CON), and the basal diet supplemented with 500 g/t or 1000 g/t IAP. Overall treatment tendencies were observed for final body weight (BW) and average daily gain (ADG) (*p* = 0.100; *p* = 0.096), with numerically higher values observed in broilers receiving 500 g/t IAP. Dietary supplementation with 500 g/t IAP significantly improved feed conversion ratio (*p* < 0.05). Across increasing dietary IAP levels, significant quadratic responses were observed for final BW, ADG, and FCR (*p* < 0.05), indicating nonlinear responses to increasing IAP supplementation levels. In addition, serum superoxide dismutase (SOD) activity was significantly increased in broilers receiving 500 g/t IAP compared with the control group (*p* < 0.05), and a significant quadratic response was observed across dietary IAP levels (*p* < 0.05). Furthermore, dietary IAP supplementation significantly reduced ileum interleukin-1β (*IL-1β*) mRNA expression in the 500 g/t group (*p* < 0.05), and *IL-1β* expression exhibited a significant quadratic response across IAP supplementation levels (*p* < 0.05). In conclusion, dietary supplementation with IAP, particularly at 500 g/t, improved feed efficiency, increased serum SOD activity, and reduced ileal *IL-1β* mRNA expression in broilers without compromising carcass characteristics or meat quality. These findings indicated that IAP has potential as a functional feed additive for further evaluation in broiler production.

## Simple Summary

Modern poultry production requires nutritional strategies that can improve broiler health and production efficiency while reducing reliance on antibiotics. Bioactive peptides are natural compounds that may help regulate antioxidant defense, immune balance, and intestinal health, but their use in broiler diets remains insufficiently studied. This study evaluated the effects of immunologically active peptides on growth performance, carcass traits, meat quality, antioxidant status, immune function, and intestinal inflammation in broiler chickens. Broilers were fed a basal diet or diets supplemented with two levels of these peptides for 42 days. The results showed that the moderate supplementation level improved feed efficiency, increased the activity of an important antioxidant enzyme in the blood, and reduced the expression of a pro-inflammatory marker in the ileum. No negative effects were observed on carcass traits or meat quality. These findings suggest that immunologically active peptides may be a promising natural feed additive for improving broiler health and production efficiency, supporting more sustainable poultry production.

## 1. Introduction

Modern broiler production is characterized by rapid growth rates, high stocking density, and intensive feeding systems, which may increase the susceptibility of birds to oxidative stress, inflammatory responses, immune dysregulation, and intestinal health disorders [1]. These challenges can impair nutrient utilization, compromise growth performance and meat quality, and increase disease risk [2]. Environmental stressors, such as heat stress, may exacerbate these disturbances and compromise broiler welfare [3,4,5]. However, heat stress was not imposed in the present study, and the present findings should not be extrapolated to heat-stressed broilers. With the restriction or prohibition of antibiotic growth promoters, safe and effective functional feed additives are urgently needed to support growth performance, antioxidant defense, immune homeostasis, and intestinal health in broiler chickens [6,7]. Therefore, bioactive substances from natural medicinal resources may provide a potential strategy for developing functional feed additives for broiler production.

Among natural medicinal resources, *Cicadae Periostracum* (CP), the exuviae molted from the nymph of *Cryptotympana pustulata* Fabricius, has long been used as a traditional Chinese medicinal material [8]. Traditionally, CP has been used in humans to relieve fever and sore throat, promote rash eruption, and alleviate spasms associated with pediatric convulsions [9,10,11,12]. Modern pharmacological studies have further demonstrated that CP exhibits multiple biological activities, including anti-inflammatory, antioxidant, anticonvulsive, antiallergic, and immunomodulatory effects [13,14,15,16,17]. For example, CP extracts have been reported to suppress dendritic cell activation and maturation and attenuate contact hypersensitivity responses in mice [14]. In addition, CP extracts may protect cells against oxidative injury by modulating reactive oxygen species (ROS) production, interleukin-6 (*IL-6*) expression, matrix metalloproteinase (MMP) generation, antioxidant enzyme activity, and related cellular signaling pathways [18]. It has also been reported that the extracts of CP have antioxidant, anti-inflammatory, and spasmolytic bioactivities [19]. These findings relate to crude CP extracts and therefore do not establish that the reported activities are specifically attributable to their peptide fraction.

Bioactive peptides are amino acid sequences that exhibit antioxidant, anti-inflammatory, antimicrobial, and immunomodulatory activities beyond their basic nutritional value [20,21]. In recent years, bioactive peptides derived from natural sources have gained increasing interest as potential feed additives in livestock production due to their ability to improve growth performance, enhance immune function, and modulate antioxidant status [22,23,24]. Various studies in poultry have demonstrated that dietary supplementation with bioactive peptides can positively affect growth performance, nutrient digestibility, protein metabolism, antioxidant capacity, and immune responses [25,26,27]. However, most of these studies have utilized peptides derived from plant sources such as soybean, cottonseed, or from microorganisms via fermentation [28,29,30,31,32]. For example, bioactive peptides derived from solid-state fermented cottonseed meal improved average daily gain, crude protein digestibility, serum immunoglobulin concentrations, and antioxidant-related indices in yellow-feathered broilers [25]. Compared with plant- and microbial-derived peptides, insect-derived bioactive compounds have received relatively less attention in poultry nutrition, although insect-derived antimicrobial peptides and related bioactive components have been reported to exert antimicrobial and immunomodulatory effects [33,34,35]. Further investigation is therefore required to determine whether peptide preparations obtained from alternative insect materials can be used in broiler diets.

Although crude CP extracts and bioactive peptides from other sources have been investigated separately, information regarding the dietary application of CP-derived peptide preparations in broilers remains limited. Therefore, the present study was conducted to evaluate the effects of dietary IAP supplementation in broilers at two inclusion levels, 500 and 1000 g/t, corresponding to 0.05% and 0.10% of the diet, respectively. These levels were selected based on our preliminary test and economic consideration. The findings of this study may provide a scientific basis for the potential application of CP-derived IAP as a functional feed additive in broiler production.

## 2. Materials and Methods

### 2.1. Experimental Design and Animal Management

A total of 180 healthy one-day-old male Ross 308 broilers with an initial body weight of approximately 48 g were randomly allocated to 3 dietary treatments, with 6 replicate cages per treatment and 10 birds per cage. The treatments consisted of a corn–soybean meal basal diet (control) or a basal diet supplemented with 500 (IAP500) or 1000 g/t (IAP1000) immunologically active peptides (IAP). The IAP product was derived from *Cicadae Periostracum* and provided by Shandong Kunhe Biotechnology Co., Ltd. (Zaozhuang, China), and the carrier was maifan stone. Firstly, a cicada-derived molt peptide library was constructed using bioinformatics methods, and virtual screening of immune active peptides was performed using neural network prediction analysis and molecular docking techniques. Secondly, high-potential candidate peptides were chemically synthesized using solid-phase peptide synthesis technology, and the target peptide segment CS6013 with amino acid sequence MVVMSTTMMIVVLVMLVMMLIVVVVVVMMMAE and molecular weight 3574.81 Da was determined based on comprehensive activity intensity and stability indicators. Thirdly, based on the reverse translation of the sequence, the corresponding DNA coding sequence was designed and cloned into an expression vector after codon optimization. Finally, the recombinant engineering strain was constructed by transforming it into engineered lactic acid bacteria, and large-scale fermentation production of CS6013 was achieved through expanded cultivation and induced expression. The feeding program comprised starter (d 1–14), grower (d 15–28), and finisher (d 29–42) phases. The mash-form basal diets were formulated according to the nutrient specifications for Ross 308 broilers [36], and their ingredient composition and calculated nutrient levels are presented in Table 1.

The birds were housed in three-tier stainless-steel cages (120 cm × 80 cm × 45 cm per cage) equipped with trough feeders and nipple drinkers. Feed and fresh water were provided ad libitum, and the feeding and drinking systems were inspected daily to ensure unrestricted access. Ambient temperature was maintained at 31–33 °C during the first week and at approximately 29.5 °C during the second week. The temperature was gradually reduced to 22–24 °C and maintained within this range until the end of the experiment. Relative humidity was maintained at 60–65%. Additionally, 24 h lighting was provided during the first 3 d, followed by a 23 h light and 1 h dark photoperiod from d 4 to 42. The cages were cleaned daily, and the excreta trays were removed and sanitized regularly.

No vaccines, antibiotics, or therapeutic medications were administered during the experimental period. All chicks were obtained from a commercial hatchery with health certification, and no clinical signs of disease were observed throughout the trial. Bird health was assessed twice daily by trained personnel based on general appearance, behavior, clinical signs, feed and water consumption, and mortality. Culling occurred because of the abnormal light weight.

### 2.2. Growth Performance

On days 1, 14, 28, and 42 of the experiment, broilers were weighed by replicate, and body weight (BW) was recorded. Feed offered and residual feed in each replicate were weighed and recorded throughout the experimental period to determine feed intake. Average daily gain (ADG), average daily feed intake (ADFI), and feed conversion ratio (FCR) were calculated for each growth phase, including days 1–14, 15–28, 29–42, and the overall period of days 1–42. In case of mortality, the body weight of dead birds and the remaining feed were recorded, and growth performance data were corrected accordingly. Growth performance calculations were corrected using the body weights of dead or culled birds and the corresponding feed consumption.

### 2.3. Sample Collection

On day 42 of the trial, one bird per replicate with body weight close to the pen average was selected. Blood samples were collected from the wing vein into serum separator tubes before euthanasia. The samples were allowed to clot at room temperature for 30 min and centrifuged at 3000× *g* for 10 min. The serum was separated and stored at −20 °C for analysis. The birds were then euthanized by manual cervical dislocation performed by trained personnel, then exsanguinated by severing the major cervical blood vessels. Death was confirmed by the absence of rhythmic breathing, corneal reflex, and heartbeat before dissection.

The ileum mucosal samples were gently scraped from the ileum wall, immediately snap-frozen in liquid nitrogen, and stored at −80 °C for subsequent analysis of inflammatory gene expression. Following exsanguination, the eviscerated carcass, *pectoralis major*, *pectoralis minor*, leg muscle, and abdominal fat were dissected and weighed for the determination of carcass traits. Samples of the pectoralis major were collected for the measurement of pH at 45 min and 24 h postmortem, drip loss, cooking loss, shear force, and meat color.

### 2.4. Determination of Carcass Traits

Carcass traits were measured following the method described by Park et al. [37]. At 42 days of age, one bird close to the average body weight per replicate was fasted overnight and then euthanized by manual cervical dislocation performed by trained personnel, followed by exsanguination, as described in Section 2.3. After removing feathers, head, feet, and visceral organs, the eviscerated carcass weight was recorded immediately. The breast muscle (*pectoralis major* and *pectoralis minor*), leg muscle, and abdominal fat were separated and weighed accurately. The percentages of eviscerated yield, breast muscle yield, leg muscle yield, and abdominal fat yield were calculated based on the live weight at slaughter to evaluate the carcass performance of broilers.

### 2.5. Measurement of Meat Quality Attributes

Meat quality was determined according to the method of Wu et al. [38] with minor modifications. Briefly, pH values of breast muscle were measured at 45 min and 24 h postmortem using a pH-STAR pH meter (Matthäus GmbH & Co. KG, Eckelsheim, Germany). For each sample, three measurements were taken at the upper, middle, and lower positions, and the average value was recorded. Meat lightness was measured at 45 min and 24 h postmortem using an OPTO-STAR meat colorimeter (Matthäus GmbH & Co. KG, Eckelsheim, Germany). Three readings were taken at different locations on each sample and averaged. The lightness value reported by the instrument was used for meat color evaluation, because the instrument does not provide the complete CIE Lab color coordinates, redness (a*) and yellowness (b*) were not determined. For drip loss, a meat sample (5 cm × 3 cm × 2 cm) was cut perpendicular to the muscle fiber direction, weighed, and suspended by a wire hook in a sealed plastic bag at 4 °C with fibers oriented vertically downward. After 24 h, the sample was removed, blotted dry with filter paper, and reweighed. Drip loss was calculated as the percentage of weight loss relative to the initial weight. Pressing loss was determined using uniformly shaped *pectoralis major* samples (9 ± 2 g). Each sample was sealed in a plastic bag and stored at 4 °C for 24 h. After storage, surface exudate was gently removed with filter paper, and the sample was weighed (W_1_). The sample was then compressed at 30 N for 5 min using a water-holding capacity tester (RH-1000, Guangzhou Runhu Instrument Co., Ltd., Guangzhou, China) and reweighed (W_2_). Pressing loss was calculated as follows: pressing loss (%) = [(W_1_ − W_2_)/W_1_] × 100. Cooking loss was measured as follows: a trimmed breast muscle sample was stored at 4 °C for 24 h, then kept at room temperature for 30 min, weighed, and placed in a sealed plastic bag. The bag was immersed in an 80 °C water bath until the internal temperature of the sample reached 70 °C. After cooling for 30 min, the sample was removed, blotted dry, and reweighed. Cooking loss was expressed as the percentage of weight loss relative to the initial weight. Following cooking loss determination, the samples were cut parallel to the muscle fibers into three strips measuring 3 × 1 × 1 cm. Each strip was sheared once perpendicular to the muscle fibers using a texture analyzer (TA.XT Plus, Stable Micro Systems Ltd., Godalming, UK) equipped with a V-shaped blade (HDP/BSW probe). The pretest, test, and posttest speeds were 2.0, 2.0, and 10.0 mm/s, respectively, and the trigger force was 5 g. The maximum shear force was recorded for each strip, and the mean of three technical replicates was expressed in Newtons (N).

### 2.6. Assay of Serum Antioxidant Indices

Serum catalase (CAT) activity, superoxide dismutase (SOD) activity, and malondialdehyde (MDA) content were determined spectrophotometrically using commercial assay kits (catalog Nos. A007-1-1, A001-3-2, and A003-1-1, respectively; Nanjing Jiancheng Bioengineering Institute, Nanjing, China) according to the manufacturer’s instructions. CAT activity was determined using the ammonium molybdate method at 405 nm, SOD activity using the WST-1 method at 450 nm, and MDA content using the thiobarbituric acid method at 532 nm. Absorbance was measured at the corresponding wavelengths using a microplate photometer equipped with the appropriate filters (Multiskan FC, Thermo Fisher Scientific, Waltham, MA, USA).

### 2.7. Serum Immunoglobulin Measurement

Serum immunoglobulin G (IgG) and immunoglobulin A (IgA) contents were measured using commercial ELISA kits according to the manufacturer’s instructions (Nanjing Jiancheng Bioengineering Institute, Nanjing, China). Standard curves were generated using serial dilutions of the purified chicken immunoglobulin standard. The intra-assay coefficients of variation for IgG and IgA were both less than 8.0%, and the interassay coefficients of variation for IgG and IgA were both less than 10.0%. The results were expressed in g/L (for IgG) and μg/mL (for IgA) based on the standard curve.

### 2.8. Quantitative Real-Time PCR

The quantitative real-time PCR (qRT-PCR) procedures were performed according to the method described by Biswas et al. [39] with minor modifications. Briefly, total RNA was extracted from ileum mucosa samples using TRIzol reagent (Thermo Fisher Scientific, Waltham, MA, USA) following the manufacturer’s instructions. The concentration and purity of RNA were verified using a microplate reader (Thermo Fisher Scientific, Waltham, MA, USA), and the OD260/OD280 ratio of all samples ranged from 1.8 to 2.0. First-strand complementary DNA (cDNA) was synthesized using a commercial reverse transcription kit (i-presci, Sci-Tech, Beijing, China). Primer specificity was confirmed by NCBI BLAST+ 2.17.0+ alignment, and beta-actin (*β-actin*) was used as the internal reference gene for normalization of target gene expression. The primer sequences used in this study are shown in Table 2. Quantitative real-time PCR was performed on a real-time fluorescence quantitative PCR system using SYBR Green PCR Master Mix (Bio-Rad Laboratories, Inc., Hercules, CA, USA). The amplification specificity of each primer pair was confirmed by melting-curve analysis, and no-template controls were included in each run to monitor potential contamination. Each sample was analyzed in duplicate technical replicates, and the mean Ct value was used for subsequent analysis. The mRNA expression levels of interleukin-1β (*IL-1β*), interleukin-6 (*IL-6*), and interleukin-10 (*IL-10*) were determined. The relative gene expression levels were calculated using the 2^−ΔΔCT^ method [40] and normalized to the expression of *β-actin*.

### 2.9. Statistical Analysis

Data were expressed as mean ± standard error of the mean (SEM). The cage was considered as the experimental unit for all parameters. For meat quality, serum indices and ileum inflammatory parameters, one bird was randomly selected from each replicate cage for sample collection, and the measured values were analyzed based on the corresponding replicate cage. One-way analysis of variance (ANOVA) was performed using SAS software (version 9.2; SAS Inst. Inc., Cary, NY, USA). Treatment comparisons were performed using Tukey’s honestly significant difference test for multiple testing. Polynomial contrasts were performed to evaluate linear and quadratic responses to increasing dietary IAP levels. *p* < 0.05 was considered significant, and 0.05 ≤ *p* < 0.10 was considered a tendency.

## 3. Results

### 3.1. Growth Performance

The effects of dietary IAP supplementation on broiler growth performance are presented in Table 3. No significant differences were observed among treatments in initial BW or growth performance during the starter (d 1–14) and grower (d 15–28) phases (*p* > 0.05). Tendencies toward overall treatment effects were observed for final BW and ADG during the entire experimental period (*p* = 0.099 and *p* = 0.096, respectively). A significant quadratic response was observed for final BW across dietary IAP levels (*p* < 0.05). During d 29–42, FCR showed a tendency toward an overall treatment effect (*p* = 0.090), whereas the quadratic contrast also approached significant (*p* = 0.061). For the overall experimental period, dietary treatment significantly affected FCR (*p* < 0.05), with a significant quadratic contrast (*p* < 0.05); Tukey’s test showed that FCR was lower in the IAP500 group than in the control group (*p* < 0.05). No significant treatment effects were detected for ADFI during any experimental period (*p* > 0.05). During the 42 d experiment, mortality and culling ratio was 13.33% (6 death and 2 culled) in the control group, 5.00% (2 death and 1 culled) in the IAP500 group, and 10.00% (4 death and 2 culled) in the IAP1000 group.

### 3.2. Meat Quality and Carcass Traits

The effects of dietary IAP supplementation on meat quality and carcass traits of broilers are presented in Table 4 and Table 5, respectively. No significant overall treatment effects were detected for most meat quality parameters. The overall ANOVA indicated a tendency toward a treatment effect for pH at 24 h (*p* = 0.058), while a significant linear decrease was observed with increasing IAP inclusion (*p* < 0.05). The change in pH from 45 min to 24 h postmortem also showed a significant linear with increasing IAP inclusion (*p* < 0.05), although the overall treatment effect was not significant (*p* = 0.117). No significant overall treatment effects or polynomial responses were observed for drip loss, cooking loss, pressing loss, shear force, or meat color (*p* > 0.05). In addition, no significant differences were observed in eviscerated carcass weight, *pectoralis major* weight, *pectoralis minor* weight, leg muscle weight, abdominal fat weight, or their corresponding yields among the three groups (*p* > 0.05).

### 3.3. Serum Immune and Antioxidant Indices

**Figure 1:**
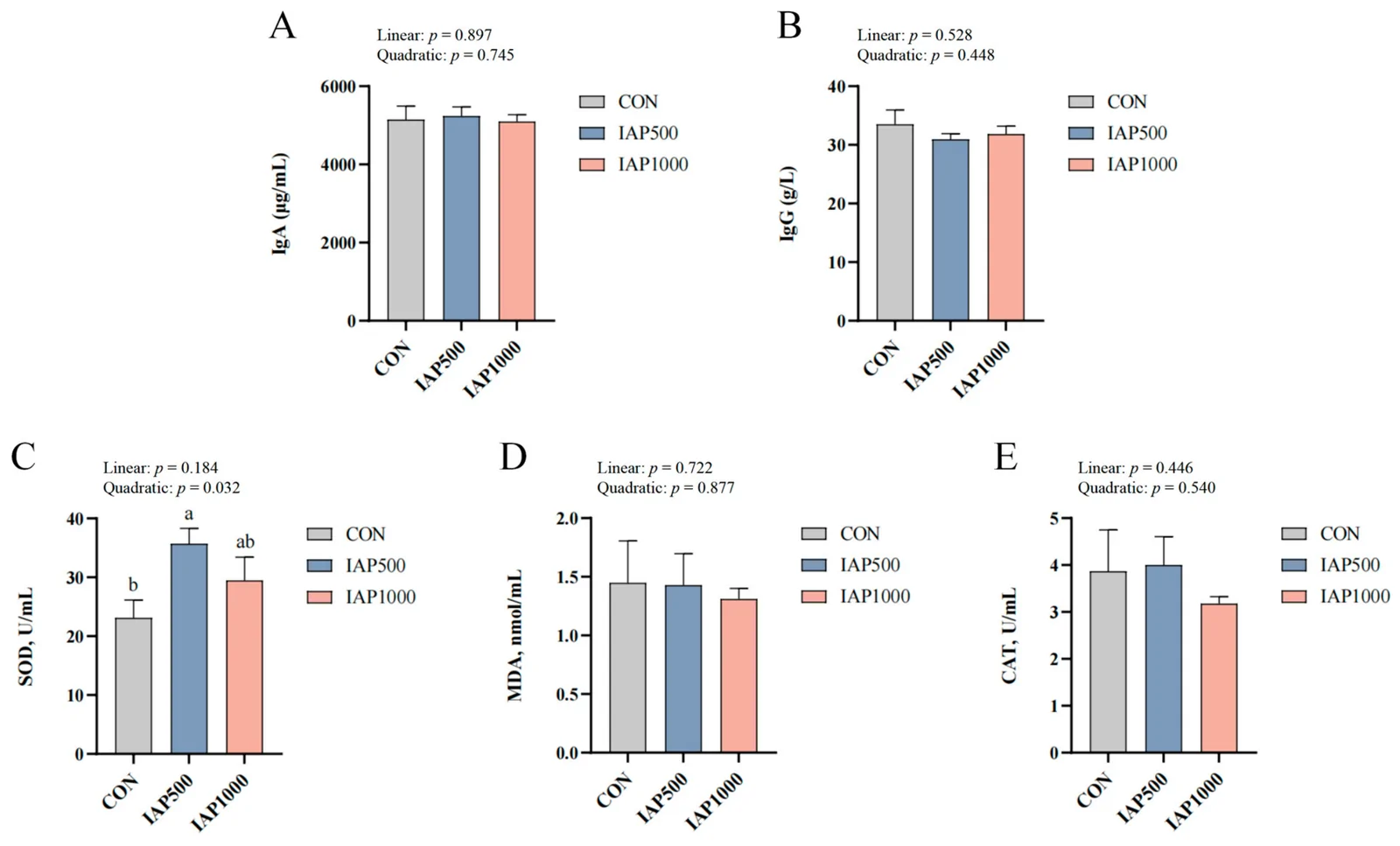
The effects of different doses of IAP supplementation on serum immunoglobulin and antioxidant indices of broilers at day 42. (**A**) Immunoglobulin A (IgA) concentration; (**B**) Immunoglobulin G (IgG) concentration; (**C**) superoxide dismutase (SOD) activity; (**D**) malondiadehyde (MDA) concentration; and (**E**) catalase (CAT) activity. Data are presented as the mean ± standard error (*n* = 6). CON: basal diet; IAP 500 and IAP 1000 groups: basal diet supplemented with 500 and 1000 g/t IAP, respectively. a, b Means listed in the same row with different superscripts are significantly different (*p* < 0.05).

The effects of dietary IAP supplementation on serum immune and antioxidant indices in broilers are presented in Figure 1. No significant differences in serum IgA and IgG concentrations were observed among treatments (*p* > 0.05, Figure 1A,B). As shown in Figure 1C–E, no significant differences in serum MDA content or CAT activity were observed among treatments (*p* > 0.05). However, broilers fed the diet supplemented with 500 g/t IAP exhibited significantly higher serum SOD activity than those in the control group (*p* < 0.05), and SOD activity showed a significant quadratic response across dietary IAP levels (*p* < 0.05).

### 3.4. Ileum Mucosal Inflammatory Gene Expression

**Figure 2:**
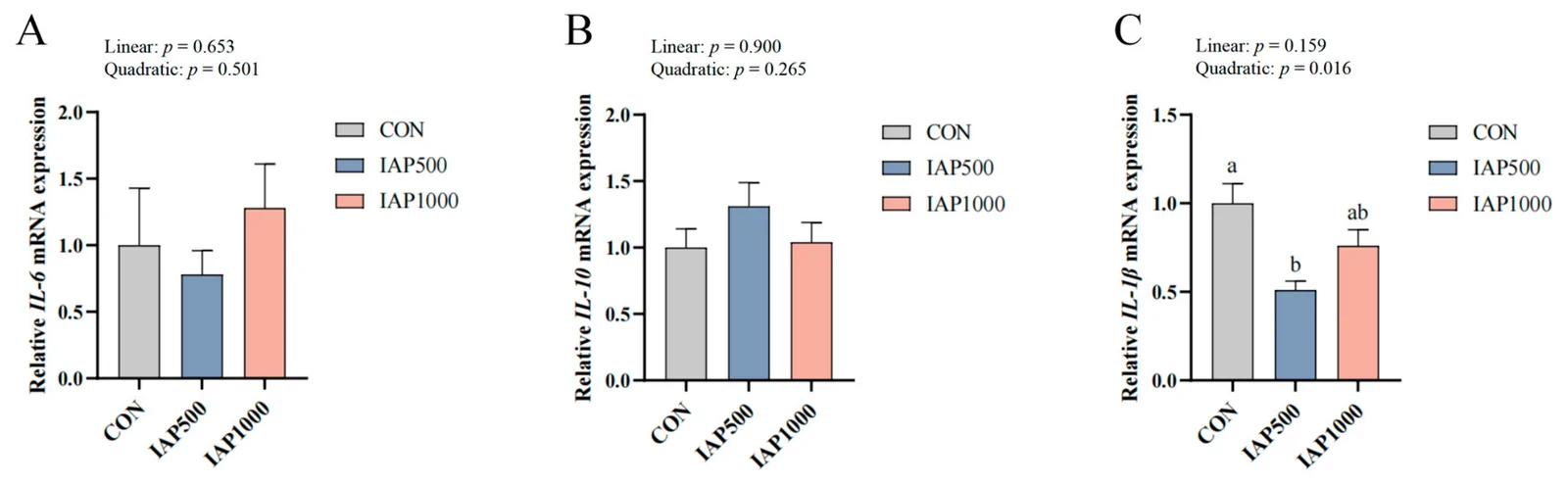
The effects of different doses of IAP supplementation on the mRNA expression of inflammatory cytokines in the ileum mucosa of broilers at day 42. (**A**) Interleukin-6 (*IL-6*) expression; (**B**) interleukin-10 (*IL-10*) expression; (**C**) interleukin-1β (*IL-1β*) expression. Data are presented as the mean ± standard error (*n* = 6). CON: basal diet; IAP 500 and IAP 1000 groups: basal diet supplemented with 500 and 1000 g/t IAP, respectively. a, b Means listed in the same row with different superscripts are significantly different (*p* < 0.05).

The effects of dietary IAP supplementation on the mRNA expression of inflammatory cytokines in the ileum mucosa of broilers are presented in Figure 2. No significant differences were observed in the relative mRNA expression levels of *IL-6* and *IL-10* among the treatment groups (*p* > 0.05). However, broilers fed the diet supplemented with 500 g/t IAP exhibited significantly downregulated mRNA expression of *IL-1β* compared with the control group (*p* < 0.05), and *IL-1β* mRNA expression showed a significant quadratic response across dietary IAP levels (*p* < 0.05).

## 4. Discussion

Bioactive peptides have attracted increasing attention as functional feed additives in poultry production because of their potential to improve growth performance [33], antioxidant defense [41], immune regulation [42], and intestinal health [43]. In the present study, dietary supplementation with IAP, particularly at 500 g/t, improved feed efficiency and was associated with changes in antioxidant and inflammation related markers without adversely affecting carcass traits or meat quality. Specifically, broilers fed 500 g/t IAP showed a significantly lower FCR, numerically higher final body weight and ADG, increased serum SOD activity, and decreased ileum *IL-1β* mRNA expression. In contrast, serum CAT activity and MDA concentration, as well as ileal *IL-6* and *IL-10* mRNA expression, were not significantly affected. These findings suggest that IAP may serve as a promising functional feed additive for improving feed efficiency and influencing selected antioxidant and inflammatory-related markers in broiler production.

Improving feed utilization efficiency is one of the primary goals in the application of functional feed additives. In the present study, dietary supplementation with 500 g/t IAP significantly improved FCR and tended to increase final body weight and ADG, whereas the 1000 g/t group did not show further improvement. Significant quadratic responses across dietary IAP levels were observed for final body weight, ADG, and FCR, suggesting that the growth-promoting effect of IAP was more evident at the moderate supplementation level. Similar beneficial effects have been reported, as IAP can improve growth performance, nutrient digestibility, antioxidant status, or feed efficiency in broilers [28,44]. Moreover, a recent meta-analysis showed that antimicrobial peptide supplementation improved ADG and FCR in broilers [45]. Among the inclusion levels tested, 500 g/t IAP was associated with the most favorable feed efficiency and numerically greater final BW and ADG. However, the significant quadratic responses observed across the three dietary levels indicate only a non-linear response pattern and are insufficient to define an optimal supplementation level. Further studies using a broader range of inclusion levels are therefore needed to establish the dose–response relationship of IAP in broilers.

Carcass traits and meat quality are important indicators for evaluating the practical application of feed additives in broiler production [46,47]. In this study, IAP supplementation had no significant effects on eviscerated carcass weight, *pectoralis major* weight, *pectoralis minor* weight, leg muscle weight, abdominal fat weight, or their corresponding yields. In addition, meat quality parameters, including pH, drip loss, cooking loss, pressing loss, shear force, and meat lightness, were not significantly affected by IAP supplementation. Although the overall treatment effect for pH at 24 h did not reach statistically significant, pH at 24 h showed a significant linear decrease with increasing IAP inclusion. Meanwhile, the change in pH from 45 min to 24 h postmortem exhibited a significant linear increase. Postmortem pH decline is an important biochemical process that can influence water-holding capacity, meat color, and other quality attributes. However, the linear changes in pH observed in the present study were not accompanied by significant changes in drip loss, cooking loss, pressing loss, shear force, or meat color, suggesting that these changes were not associated with detectable alterations in the other meat quality characteristics evaluated. Previous studies have also shown that supplementation with plant-derived or insect-derived bioactive peptides can improve productive performance without impairing carcass traits or meat quality in broilers [48,49,50]. In addition, previous studies have reported that insect-derived products, such as *Hermetia illucens* larvae meal, and other bioactive peptide sources, such as hydrolyzed black cumin seed protein, can improve productive traits without impairing carcass quality or meat quality in broilers [51,52]. Consistent with these findings, the present study showed that IAP supplementation did not adversely affect carcass characteristics or the major meat quality parameters evaluated, despite the observed linear changes in postmortem pH. These results indicate that the inclusion of IAP under the present experimental conditions did not compromise carcass or meat quality, supporting its further evaluation as a potential functional feed additive in broiler production.

Oxidative stress is associated with impaired growth, inflammatory damage, and suboptimal health status in poultry [53]. SOD is a major antioxidant enzyme involved in the conversion of superoxide radicals into hydrogen peroxide and maintaining redox homeostasis [54,55]. In the present study, serum SOD activity was significantly increased in broilers supplemented with 500 g/t IAP, whereas MDA content and CAT activity were not significantly affected. Moreover, SOD activity showed a significant quadratic response across dietary IAP levels, indicating a non-linear response pattern rather than defining an optimal supplementation level. Previous studies have reported that some insect-derived bioactive peptide preparations can influence antioxidant-related parameters in poultry [41]. However, the IAP preparation used in the present study was not chemically characterized at the individual-compound level; therefore, the observed increase in serum SOD activity cannot be attributed to specific constituents of *Cicadae Periostracum* or to a particular molecular pathway. Accordingly, this finding should be interpreted as a selective change in serum SOD activity in response to the tested IAP preparation rather than as evidence of a broad enhancement of antioxidant capacity. Further compositional characterization and mechanistic studies are needed to identify the active peptide components and clarify the mechanisms underlying this response.

Intestinal inflammatory status is closely associated with mucosal integrity, nutrient absorption, and growth efficiency in broilers [56,57]. *IL-1β* is a key pro-inflammatory cytokine involved in the initiation and amplification of inflammatory responses, and excessive *IL-1β* expression may impair intestinal barrier function [58,59,60]. In the present study, dietary supplementation with 500 g/t IAP significantly decreased ileum *IL-1β* mRNA expression. *IL-1β* mRNA expression also showed a significant quadratic response across dietary IAP levels. These findings indicate a selective transcriptional response of *IL-1β* to IAP supplementation. However, because no inflammatory or pathogenic challenge was imposed and no protein-level, histological, or intestinal barrier-related endpoints were assessed, the observed change in *IL-1β* mRNA should not be interpreted as evidence of alleviated ileal inflammation, improved barrier function, or enhanced intestinal immune homeostasis. Previous studies have reported that dietary bioactive peptides can influence cytokine expression patterns in poultry [61,62,63]. In addition, serum IgA and IgG concentrations were not significantly altered by IAP supplementation, suggesting that under normal rearing conditions, the measured responses to IAP supplementation were mainly reflected by serum SOD activity and selected ileal inflammatory related gene expression rather than systemic immunoglobulin concentrations. Further studies incorporating protein-level cytokine measurements and direct assessments of intestinal inflammatory and barrier status are needed to determine the biological significance of the observed transcriptional response.

Collectively, the primary productive response to IAP supplementation was the improvement in FCR at 500 g/t, whereas final BW and ADG only tended to increase and ADFI remained unaffected, indicating that the response was primarily attributable to enhanced feed utilization rather than increased feed intake. The increase in serum SOD activity and decrease in ileal *IL-1β* mRNA expression were concurrent, indicator specific responses; however, their contribution to the observed improvement in feed efficiency cannot be established from the present data. The significant quadratic contrasts indicate that the responses did not increase progressively across the three tested inclusion levels. Nevertheless, this pattern should not be interpreted as evidence of an optimal dose or an adverse effect of the higher inclusion level. Because the improvement in FCR at 500 g/t occurred without detrimental effects on the measured carcass traits or meat quality attributes, this inclusion level warrants further evaluation under commercial production conditions. Confirmation across a broader range of inclusion levels, together with an assessment of economic feasibility, is required before recommendations for routine application can be established.

## 5. Study Limitations

Several limitations of this study should be acknowledged. The relatively limited statistical power of the experimental design, with six replicate pens per treatment and only one bird sampled from each pen, may have limited the ability to detect treatment-related differences, particularly for variables with relatively high biological variability. The experiment was conducted using only male Ross 308 broilers maintained under a controlled cage-rearing environment, with six replicate pens per treatment and one bird sampled from each pen. These design features may limit the generalizability of the findings to other broiler populations. In addition, only three dietary inclusion levels and a single batch of the commercial IAP preparation were evaluated, and the chemical composition and batch-to-batch consistency of the product were not fully characterized. Consequently, the active constituents responsible for the observed responses and the optimal inclusion level could not be determined. Nutrient digestibility, digestive enzyme activity, intestinal morphology and barrier function, inflammatory proteins, energy metabolism, and intestinal microbiota were also not assessed, limiting mechanistic interpretation of the findings. In particular, although changes in SOD activity, IL-1β expression, and FCR were observed concurrently, the present experimental design does not permit causal relationships among these variables to be established. Future studies should therefore use chemically characterized product batches, broader inclusion levels, larger and more diverse broiler populations, and commercial production conditions, together with relevant mechanistic and economic evaluations. Given that the improvement in FCR at 500 g/t occurred without adverse effects on the measured carcass traits or meat quality characteristics, this inclusion level warrants further evaluation under commercial production conditions before routine application can be recommended.

## 6. Conclusions

In conclusion, dietary supplementation with IAP, particularly at 500 g/t, improved feed efficiency in broilers without adverse effects on carcass characteristics or meat quality. The 500 g/t supplementation level was associated with increased serum SOD activity and reduced ileal *IL-1β* mRNA expression, whereas other measured antioxidant and inflammatory-related parameters were not significantly altered. These findings suggest that IAP may have potential as a functional feed additive; however, further studies involving comprehensive biochemical, molecular, and functional assessments are required to clarify its biological mechanisms and practical application.

## Figures and Tables

**Table 1:** Ingredient and nutrient composition of the basal diet (as fed basis).

Ingredients (%)	Starter (1–14 d)			Grower (15–28 d)			Finisher (29–42 d)		
	CON	IAP500	IAP1000	CON	IAP500	IAP1000	CON	IAP500	IAP1000
Corn	50.23	50.18	50.13	53.23	53.18	53.13	54.63	54.58	54.53
Soybean meal	39.50	39.50	39.50	35.80	35.80	35.80	33.50	33.50	33.50
Wheat barn	2.50	2.50	2.50	2.50	2.50	2.50	2.50	2.50	2.50
Soybean oil	4.17	4.17	4.17	5.19	5.19	5.19	6.39	6.39	6.39
Dicalcium phosphate	0.95	0.95	0.95	0.70	0.70	0.70	0.60	0.60	0.60
Limestone	1.50	1.50	1.50	1.50	1.50	1.50	1.30	1.30	1.30
Salt	0.25	0.25	0.25	0.20	0.20	0.20	0.20	0.20	0.20
DL-Methionine	0.22	0.22	0.22	0.20	0.20	0.20	0.20	0.20	0.20
L-Lysine HCl	0.10	0.10	0.10	0.10	0.10	0.10	0.10	0.10	0.10
Premix ^1^	0.58	0.58	0.58	0.58	0.58	0.58	0.58	0.58	0.58
IAP	0	0.05	0.10	0	0.05	0.10	0	0.05	0.10
Total	100	100	100	100	100	100	100	100	100
Analyzed nutrient levels									
Crude protein, %	21.85	21.79	21.87	20.33	20.47	20.39	19.62	19.58	19.67
Calcium, %	0.90	0.89	0.89	0.82	0.84	0.83	0.71	0.72	0.73
Total phosphorus, %	0.57	0.56	0.55	0.51	0.50	0.52	0.48	0.47	0.48
Calculated nutrient levels									
ME, kcal/kg	3000	3000	3000	3100	3000	3000	3200	3000	3000
Available phosphorus, %	0.29	0.29	0.29	0.23	0.29	0.29	0.21	0.29	0.29
Lysine, %	1.31	1.31	1.31	1.21	1.31	1.31	1.15	1.31	1.31
Methionine, %	0.57	0.57	0.57	0.52	0.57	0.57	0.51	0.57	0.57
Threonine, %	0.90	0.90	0.90	0.84	0.90	0.90	0.80	0.90	0.90

^1^ Premix supplied per kg of diet: vitamin A (VA), 11,800 IU; vitamin D_3_ (VD_3_), 2500 IU; vitamin E (VE), 15 IU; vitamin K_3_ (VK_3_), 2.65 mg; vitamin B_1_ (VB_1_), 2 mg; vitamin B_2_ (VB_2_), 6 mg; vitamin B_12_ (VB_12_), 0.025 mg; biotin 0.0325 mg; folic acid 1.25 mg; calcium pantothenate, 12 m; niacin, 50 mg; copper (Cu), 8 mg; zinc (Zn), 75 mg; iron (Fe), 80 mg; manganese (Mn), 100 mg; selenium (Se), 0.15 mg; and iodine (I), 0.35 mg. ME, metabolizable energy.

**Table 2:** Primer sequences and amplification information used for quantitative real-time PCR.

Target Gene	NCBI RefSeq Accession No.	Forward Primer (5′-3′)	Reverse Primer (5′-3′)	Amplicon Length (bp)	Annealing Temp. (°C)
*IL-6*	NM_204628.2	AAATCCCTCCTCGCCAATCTGAAG	CCCTCACGGTCTTCTCCATAAACG	106	60
*IL-10*	NM_001004414.4	CAGCACCAGCCATCAGCAGAG	ATCCATCTTCTCGAACGTCTCCTTG	148	60
*IL-1β*	NM_204524.2	GCCGAGGAGCAGGGACTTTG	GAAGGACTGTGAGCGGGTGTAG	136	60
*β-actin*	NM_205518.2	TATGTGCAAGGCCGGTTTC	TGTCTTTCTGGCCCATACCAA	110	60

*IL-6*, interleukin-6; *IL-10*, interleukin-10; *IL-1β*, interleukin-1β; *β-actin*, beta-actin.

**Table 3:** Effects of dietary supplementation on growth performance in broilers.

	Level (g/t Feed)				*p*-Value		
	0	500	1000	SEM	ANOVA	Linear	Quadratic
BW, g							
d 1	48.17	48.08	48.33	0.71	0.971	0.875	0.856
d 14	375	389	386	13	0.626	0.477	0.520
d 28	1309	1408	1331	43	0.303	0.745	0.136
d 42	2412	2599	2477	62	0.099	0.439	0.046
ADG, g/d							
d 1–14	23.33	24.32	24.09	0.92	0.611	0.470	0.504
d 15–28	66.77	72.80	67.43	2.50	0.306	0.875	0.132
d 29–42	78.77	85.10	81.90	2.81	0.246	0.400	0.148
d 1–42	56.29	60.74	57.81	1.47	0.096	0.444	0.043
ADFI, g/d							
d 1–14	29.97	30.35	30.22	0.80	0.932	0.811	0.777
d 15–28	96.87	104.46	101.21	3.23	0.377	0.423	0.254
d 29–42	146.49	147.15	146.98	3.92	0.993	0.933	0.934
d 1–42	91.11	93.99	92.81	2.13	0.682	0.611	0.484
FCR							
d 1–14	1.285	1.252	1.256	0.036	0.305	0.217	0.355
d 15–28	1.457	1.435	1.504	0.023	0.183	0.215	0.164
d 29–42	1.866	1.732	1.795	0.049	0.090	0.230	0.061
d 1–42	1.620 ^b^	1.547 ^a^	1.605 ^ab^	0.024	0.019	0.533	0.006

BW, body weight; ADG, average daily gain; ADFI, average daily feed intake; FCR, feed conversion ratio. ^a, b^ Means listed in the same row with different superscripts are significantly different (*p* < 0.05).

**Table 4:** Effects of dietary supplementation on meat quality of broilers.

	Level (g/t Feed)			*p*-Value			
Item	0	500	1000	SEM	ANOVA	Linear	Quadratic
pH 45 min	6.26	5.98	6.30	0.11	0.171	0.86	0.065
pH 24 h	6.14 ^x^	5.58 ^y^	5.61 ^xy^	0.15	0.058	0.043	0.177
ΔpH	0.13	0.39	0.69	0.20	0.117	0.048	0.564
Drip loss (%)	5.40	4.62	4.63	0.39	0.388	0.24	0.483
Cooking loss (%)	13.67	13.87	13.55	0.87	0.966	0.922	0.755
Pressing loss (%)	15.15	12.17	10.61	2.41	0.476	0.238	0.828
Shear force (N)	19.05	20.69	25.36	2.05	0.627	0.348	0.877
Lightness (OPTO value) at 45 min	46.59	48.45	45.29	4.04	0.881	0.84	0.652
Lightness (OPTO value) at 24 h	54.11	56.19	62.51	3.29	0.278	0.13	0.649

ΔpH, change in pH between 45 min and 24 h postmortem; N, newtons. ^x, y^ Means listed in the same row with different superscripts are tended to be different (0.05 ≤ *p* < 0.10).

**Table 5:** Effects of dietary supplementation on carcass performance of broilers.

	Level (g/t Feed)			*p*-Value			
Item	0	500	1000	SEM	ANOVA	Linear	Quadratic
Eviscerated carcass weight (g)	1884	1963	1808	69	0.376	0.488	0.227
*Pectoralis major* weight (g)	443	438	427	20	0.871	0.618	0.891
*Pectoralis minor* weight (g)	79.61	84.03	84.21	3.26	0.520	0.261	0.983
Leg muscle weight (g)	256	276	246	11	0.255	0.579	0.123
Abdominal fat weight (g)	17.05	18.72	19.67	1.99	0.718	0.429	0.899
Eviscerated yield (%)	82.52	81.49	80.43	0.70	0.231	0.092	0.987
*Pectoralis major* yield (%)	23.45	22.34	23.84	0.47	0.208	0.903	0.082
*Pectoralis minor* yield (%)	4.23	4.18	4.71	0.19	0.179	0.126	0.282
Leg muscle yield (%)	13.55	14.10	13.68	0.53	0.780	0.879	0.498
Abdominal fat yield (%)	0.88	0.95	1.08	0.10	0.435	0.211	0.827

## Data Availability

The original contributions presented in this study are included in the article. Further inquiries can be directed to the corresponding author(s).
